# The Impact of the Measure Used to Calculate the Distance between Exchange Rate Time Series on the Topological Structure of the Currency Network

**DOI:** 10.3390/e26040279

**Published:** 2024-03-25

**Authors:** Joanna Andrzejak, Leszek J. Chmielewski, Joanna Landmesser-Rusek, Arkadiusz Orłowski

**Affiliations:** 1 Institute of Civil Engineering, Warsaw University of Life Sciences—SGGW, 02-787 Warsaw, Poland; joanna_andrzejak@sggw.edu.pl; 2 Institute of Information Technology, Warsaw University of Life Sciences—SGGW, 02-776 Warsaw, Poland; leszek_chmielewski@sggw.edu.pl; 3 Institute of Economics and Finance, Warsaw University of Life Sciences—SGGW, 02-787 Warsaw, Poland; joanna_landmesser@sggw.edu.pl

**Keywords:** exchange rates, currency network, dissimilarity measures, C58, C32, C38

## Abstract

Structural properties of the currency market were examined with the use of topological networks. Relationships between currencies were analyzed by constructing minimal spanning trees (MSTs). The dissimilarities between time series of currency returns were measured in various ways: by applying Euclidean distance, Pearson’s linear correlation coefficient, Spearman’s rank correlation coefficient, Kendall’s coefficient, partial correlation, dynamic time warping measure, and Kullback–Leibler relative entropy. For the constructed MSTs, their topological characteristics were analyzed and conclusions were drawn regarding the influence of the dissimilarity measure used. It turned out that the strength of most types of correlations was highly dependent on the choice of the numeraire currency, while partial correlations were invariant in this respect. It can be stated that a network built on the basis of partial correlations provides a more adequate illustration of pairwise relationships in the foreign exchange market. The data for quotations of 37 of the most important world currencies and four precious metals in the period from 1 January 2019 to 31 December 2022 were used. The outbreak of the COVID-19 pandemic in 2020 and Russia’s invasion of Ukraine in 2022 triggered changes in the topology of the currency network. As a result of these crises, the average distances between tree nodes decreased and the centralization of graphs increased. Our results confirm that currencies are often pegged to other currencies due to countries’ geographic locations and economic ties. The detected structures can be useful in descriptions of the currency market, can help in constructing a stable portfolio of the foreign exchange rates, and can be a valuable tool in searching for economic factors influencing specific groups of countries.

## 1. Introduction

Various relationships between different currencies can be defined and investigated. They can be used as an effective tool in studying the structures which appear in the market of currencies. The structural properties of the foreign exchange market can be fruitfully studied using topological networks. Topological networks are one of the tools for analyzing the relationships between the economies in which these currencies are used. The topology of the network is an important and relatively advanced concept that can be easily observed by human eyes, and which enables qualitative analysis of what basically is of a quantitative nature. This helps to discover the structures which emerge from the relationships. Qualitative representation of quantitative relations makes it possible to understand the important relationships occurring in the market. The changes in the topology of the market in time and the knowledge of the important events which occurred, started or ended at certain time points can also carry important information. The fundamental difference between correlation and dependency must always be kept in mind. However, the relations based on data, when visualized, make it easier to discover or postulate advanced hypotheses on the influences or dependencies which can play a role in the international market.

In this research we assessed the changes that occurred in the topological structure of the global currency market in 2020–2022. These changes were mainly caused by the COVID-19 pandemic and Russia’s invasion of Ukraine and are of the nature of global crises. The situation in currency markets is currently very volatile; hence we consciously set the time scope of our research. Data for 37 major currencies and four precious metals were used. Although the currency market and the precious metals market are separate markets, this choice was dictated by the fact that some researchers use metal prices as a base when constructing a correlation network for currencies (e.g., [1]). Due to the dynamically changing situation in global markets, the analysis was carried out in three sub-periods:

− in the year of the COVID-19 pandemic outbreak (from 1 January to 31 December 2020);
− in the next year of the pandemic (from 1 January to 31 December 2021);
− in the year of Russia’s invasion of Ukraine (from 1 January to 31 December 2022).

The starting point for building a network in which pairs of highly correlated currencies are connected is the analysis of the correlation between them, which allows for the creation of a correlation matrix. Relationships between currency pairs can be defined by various measures (usually statistical) and analyzed using various algorithms. The selection of these measures and algorithms can and does influence the topology of the resulting network. In this paper we wish to present an approach to studying this issue based on the use of minimum spanning trees (MSTs). The topological features of the resulting trees will be further analyzed. They will then be compared among themselves and conclusions will be drawn regarding the impact of the distance measure used on the final result. In our research we will refer to the thesis of Basnarkov et al. (2019) [2], which states that the strength of ordinary correlations is highly dependent on the choice of numeraire currency, while partial correlations are invariant in this aspect.

In the study, the following research questions were posed:

1. What is the impact of the distance measure used on the topological structure of the currency network?
2. Are different measures equally sensitive to the choice of numeraire currency?
3. Does the topological structure of the currency network during the periods of the pandemic crisis and the crisis caused by Russia’s invasion of Ukraine differ from the structure of the network in the period in between?

This paper is organized as follows. The state of the art of the subject is given in Section 2 in the form of a review of relevant publications. Data used are thoroughly described in Section 3. In Section 4 we present a detailed description of our research methods, including precise definitions of all the relevant concepts used. The main results of our investigations are presented in Section 5. We finish in Section 6 with the conclusions.

## 2. Literature Review

In this work, we focus on the analysis of a complex system that consists of many components which interact with each other. The pattern of connections between components is crucial to the behavior of the system [3]. A system cannot be understood by examining its parts in isolation. A popular way to study the pattern of interactions in a given system is to construct a network (graph) with nodes and edges [4,5]. The study of networks is the domain of graph theory, the beginning of which is considered to be the publication of Euler’s solution to the Königsberg bridge problem in 1736. After the initial descriptions of regular graphs with usually a small number of nodes, theorists became interested in random graphs [6]. The groundbreaking works of Watts and Strogatz (1998) [7] and Barabasi and Albert (1999) [8], along with the rapid development of computerization, triggered the intensive development of network science. In contrast to the limited sizes of graphs analyzed so far, it was noticed that real-world networks often manifest a *small-world* phenomenon [9] and high levels of clustering with power-law distribution for the degree of vertices.

Much research work has focused on the topological properties of networks, particularly those relating to possible connections between nodes. Topology is important for the network’s resilience to external perturbations, like random failures or targeted attacks [10,11]. It also plays a role in the propagation of processes operating on the network (e.g., disease spread, information flow; see Liu et al. (2003) [12]). The observed heterogeneity in the strength of interactions between components led to the study of weighted networks. In addition, the local edge distribution resulted in the consideration of the network’s community structure [13,14,15]. The structure of a network determines how vertices are connected and how data is transferred between them. Minimal spanning trees (MSTs), introduced by Kruskal (1956) [16], compress information about the global network structure and simplify the analysis by reducing the number of elements to be compared. Although most of the early network research focused on networks at a fixed point in time, many different approaches were later adopted to construct and analyze the structural dynamics of networks (e.g., Palla et al. (2007) [17]).

Currently, many researchers are focusing on financial markets, which are often considered as evolving complex systems. The most common application of the network in the financial market is to investigate the relationship between time series of financial asset prices [18,19,20]. In the networks built for this aim, each node represents an asset, and each weighted edge represents a correlation between time series of asset prices.

In our work, we will study FX market networks in which each vertex represents an exchange rate and the edges represent the correlations between them. Topological networks of currencies have been studied previously, e.g., by McDonald et al. (2005) [21], Ortega and Matesanz (2006) [22], Naylor et al. (2007) [23], Górski et al. (2008) [1], or Kwapień et al. (2009) [24]. The algorithm used to reveal relationships between currency pairs is usually the MST algorithm. Ortega and Matesanz (2006) [22], for example, analyzed the effects of currency crises using time series of exchange rates for 28 countries. Naylor et al. (2007) [23] used MSTs to extract a topological map for 44 currencies and generated a robust scale-free network, of which the topology proved to be stable even during crises. Exchange rate correlation matrices for 60 currencies were examined by Górski et al. (2008) [1], who presented MSTs with inverse power scaling. Kwapień et al. (2009) [24] studied the topology of the foreign exchange market based on the exchange rates of 46 currencies from 1998–2008 and constructed different currency networks depending on the choice of base currency. Their structure was not stable, but there were clusters that persisted over time.

Many researchers have focused on the structural evolution of the foreign exchange market during periods of crisis. Jang et al. (2011) [25] studied the characteristics of the foreign exchange market from 1990 to 2008. They noted that the average correlation coefficient between currencies decreased during crises, while the length of the tree increased. The EUR and USD showed a strong negative correlation after the Southeast Asian crisis in 1997. Asian and Latin American currencies moved away from the cluster center (USD). Feng and Wang (2010) [26] found that the correlation between Asian currencies and the US dollar was weakening, while intra-Asian interactions were intensifying. However, Wang et al. (2013) [27] pointed out the instability of the Asian cluster. Wang et al. (2012) [28] divided the 2005–2011 analysis period into three sub-periods: before, during, and after the US subprime crisis, and the changes in the MST structure were analyzed. The authors confirmed that the USD and EUR were still the dominant global currencies, but the dollar gradually lost its central position, while the euro acted as a stable center throughout the crisis. In contrast, two years later, Wang et al. (2014) [29] found that the financial crisis resulted in a more central position for the USD. Structural changes in the currency network before, during, and after the subprime crisis were also studied by Kazemilari and Mohamadi (2018) [30], and their analysis showed significant changes in the constructed MSTs. Rešovský et al. (2013) [31] studied the linkages of major world and European currencies and proved the important position of the currencies of Hong Kong and Singapore, and in Central Europe of the Polish zloty. In the study of Limas (2019) [32], the Latin American currencies were studied, uncovering hierarchical structures. The community structure of the international currency network was confirmed by Fenn (2010) [15] and Cao et al. (2020) [33]. Miśkiewicz (2021) [34] found that during crises, exchange rate correlations increase, causing more cliques and higher ranks for nodes in the network.

Our study will use data from the 2020–2022 period for the quotations of the world’s major currencies, and four precious metals which play a role similar to that of the currencies, in some sense. During this period, the currency market was particularly affected by the COVID-19 pandemic (cf. [35,36,37]) and Russia’s invasion of Ukraine.

Research on the evolution of the currency network in the context of COVID-19 is scarce. One example is a study by Gupta and Chatterjee (2020) [38], which proposed a measure based on the lead–lag relationship to analyze the relationship between currencies. They noted that as the crisis progressed, the 29 currencies analyzed became strongly interconnected, with the USD was taking a position closer to the center of the currency network considered. Also, the outbreak of the Russian–Ukrainian war in February 2022 had a strong impact on the foreign exchange markets (cf. [39,40]). Complex network theory was applied to analyze the relationship between global stock markets during the Russia–Ukraine war in Zaheer et al. (2024) [41], but to the authors’ knowledge, no one has studied the impact of the Russian invasion of Ukraine on the topological structure of the global currency network to date.

The starting point for network construction algorithms is the concept of the distance between the objects under study, which can be defined in various ways for properly represented data. In this study, we shall consider the distance as a measure of dissimilarity, which does not necessarily have properties of the distance in the strict mathematical sense. Therefore, we shall also use the notion of *distance measure*. Since the main purpose of our analysis is to study the impact of the applied distance measures between exchange rate time series on the topological structure of the foreign exchange market, we examined what has been proposed so far in the literature in this regard (cf. Marti et al., 2021 [42]). In most analyses of network evolution, Pearson’s linear correlation is chosen as the preferred metric [19,23,25,43], but there are alternative measures for this (cf. Aghabozorgi et al. (2015) [44]). Distances between time series vectors can also be determined using the Euclidean measure, Spearman’s rank correlation coefficients, Kendall’s coefficients, partial correlations, or more ambitiously using the copula functions, the Granger causality distance, the PCA distance, dynamic time warping (DTW) or entropy measures (e.g., Kullback–Leibler or Jensen–Shannon divergence). A good illustration of the use of the partial correlation coefficient to build a network are the works of Kenett et al. (2010) [45] and Basnarkov et al. (2019) [2]. The copula-based approach, which aims at including non-linear relationships in the analysis, was used by Marti et al. (2016) [46]. The Granger causality distance constitutes an attempt to quantify how one financial instrument provides information about another instrument (see Billio et al., 2012 [47]). An example of the use of PCA distance is the work of Fenn (2010) [15]. The DTW distance, which is common in fields such as biometrics and computer animation, was used to study the topology of the currency network by Wang et al. (2012) [28] and Gupta and Chatterjee (2020) [38]. In Shternshis et al. (2022) [48], the clustering of financial time series into different groups according to the Kullback–Leibler entropy measure was performed. Chakraborty et al. (2020) [49] examined the relationship between currencies and analyzed network clusters during periods of severe international crises using a method based on the Jensen–Shannon divergence. The variety of instruments that have been previously used in network analyses justifies the intention to compare the effects of their use, which is what is done in this work.

An additional problem in foreign exchange research is the choice of base currency (*numéraire*, or simply numeraire). Currencies are priced against each other, so there is no independent numeraire (Keskin et al., 2011 [50]). Any currency selected as a base will be excluded from the network, but its internal patterns may indirectly influence the overall patterns. Different choices will yield different conclusions if strong cross-correlations exist between currencies. This problem has no standard solution (cf. [23,28,38]). Sometimes the most traded asset is taken as numeraire, while another less liquid asset is used to compare the results [2]. Moreover, according to [2], the choice of the numeraire currency strongly affects the strength of Pearson’s linear correlation, while partial correlations are invariant in this aspect (as we have already mentioned in the Introduction). In our study of the characteristics of the currency market, we have considered all the possible assets as numeraires in the calculations. Only when visualizing the constructed trees did we limit ourselves to one reference currency, for technical reasons. A natural choice for the authors of the article was the PLN—the domestic currency.

## 3. Description of Data

The analysis was based on data for the 1 January 2020–31 December 2022 period and three sub-periods: 1 January–31 December 2020 (the year of the COVID-19 pandemic outbreak), 1 January–31 December 2021, and 1 January–31 December 2022 (the year of Russia’s invasion of Ukraine). Daily data on exchange rates for 37 currencies and prices for four precious metals were obtained from the https://stooq.pl/service (accessed on 15 January 2023). A list of the currencies and metals studied, along with their abbreviations, is shown in Table 1. The descriptive statistics for the analyzed time series measured in PLN, in the whole period 1 January 2020–31 December 2022, are collected in Table 2.

The series were smoothed using a 5-day moving average. Our preliminary research results showed that the exchange rates of the analyzed currencies were not stationary (in the ADF test, there was no grounds to reject the null hypothesis of non-stationarity) and the daily price changes were not distributed normally. At the same time, as expected, the series of logarithmic rates of return were stationary. Therefore, logarithmic rates of return were used in further analysis.

## 4. Description of the Research Method

### 4.1. Minimum Spanning Trees

In the paper, the topological network analysis is used to discover the structural properties of the foreign exchange market. A network is a graph consisting of nodes (vertices) and edges. In a formal way, an undirected graph G is a pair G=(V,E), where V is a set of nodes and E is a set of edges, $E\subseteq \left\{\left(u,v\right):u,v\in V\right\}$. A weighted graph G=(V,E,w) is a graph in which each edge is assigned a non-negative weight.

A tree is an undirected graph that is connected and acyclic, i.e., there is a path for each node to any other node and it is the only possible path between them. The spanning tree of a graph G=(V,E) is a tree that has all the vertices from set V, and the set of edges of the tree is a subset of set E. For a weighted, undirected graph G=(V,E,w), a minimum spanning tree (MST) is a spanning tree T for which the sum of the weights of all edges is minimal. In this work we use the node-based greedy Prim’s algorithm to find the MST (Prim, 1957 [51]).

### 4.2. Determining the Distance between Currency Nodes

Consider a set of random variables $V=\left\{X_{1},X_{2},\ldots ,X_{m}\right\}$ (a set of return series for exchange rates in our case). The goal is to construct a minimum spanning tree (MST) for these variables. The size of dissimilarity between two time series $X_{i}=(x_{i1},\ldots ,x_{iT})$ and $X_{j}=(x_{j1},\ldots ,x_{jT})$ could be determined using the following measures.

1. Euclidean distance $d_{e}$:
  $$d_{e}\left(X_{i},X_{j}\right)=\sqrt{\sum_{t=1}^{T}{\left(x_{it}-x_{jt}\right)}^{2}}.$$
2. Distance $d_{p}$ based on Pearson’s correlation coefficient $\rho_{p}$:
  $$d_{p}\left(X_{i},X_{j}\right)=\sqrt{2[1-\rho_{p}\left(X_{i},X_{j}\right)}]\in [0,2],$$
  $$\rho_{p}\left(X_{i},X_{j}\right)=\frac{\sum_{t=1}^{T}\left(x_{it}-{\bar{x}}_{i})(x_{jt}-{\bar{x}}_{j}\right)}{\sqrt{\sum_{t=1}^{T}{\left(x_{it}-{\bar{x}}_{i}\right)}^{2}}\sqrt{\sum_{t=1}^{T}{\left(x_{jt}-{\bar{x}}_{j}\right)}^{2}}}\in [-1,1],$$
  where ${\bar{x}}_{i}$ and ${\bar{x}}_{j}$ are the average values of the series examined.
3. Distance $d_{s}$ based on Spearman’s correlation coefficient $\rho_{s}$ (rank-based correlation coefficient):
  $$d_{s}\left(X_{i},X_{j}\right)=\sqrt{2[1-\rho_{s}\left(X_{i},X_{j}\right)}]\in [0,2],$$
  $$\rho_{s}\left(X_{i},X_{j}\right)=1-\frac{6\sum_{t=1}^{T}d_{t}^{2}}{T\left(T^{2}-1\right)}\in [-1,1],$$
  where $d_{i}$ is the difference in the ranks given to the two variables values.
4. Distance $d_{k}$ based on Kendall’s correlation coefficient τ, which measures the correspondence between the rankings of two variables:
  $$d_{k}\left(X_{i},X_{j}\right)=\sqrt{2[1-\tau \left(X_{i},X_{j}\right)}]\in [0,2],$$
  $$\tau \left(X_{i},X_{j}\right)=\frac{N_{c}-N_{d}}{\frac{1}{2}n(n-1)}\in [-1,1],$$
  where $N_{c}$—number of concordant pairs, $N_{d}$—number of discordant pairs. A pair is concordant if the observation that ranks higher for variable $X_{i}$ is also higher for variable $X_{j}$. A pair is discordant if the observation that ranks higher for $X_{i}$ is lower for $X_{j}$.
5. Distance $d_{pcorr}$ based on the partial correlation coefficient $\rho_{pcorr}$, which measures the degree of association between variables, with the influence of a set of controlling variables removed:
  $$d_{pcorr}\left(X_{i},X_{j}\right)=\sqrt{2[1-\rho_{pcorr}}]\in [0,2],$$
  $${\rho_{pcorr}=\rho }_{X_{i}X_{j}.V\backslash \{X_{i},X_{j}\}}=-\frac{p_{ij}}{\sqrt{p_{ii}p_{jj}}},$$
  where $\rho_{X_{i}X_{j}.V\backslash \left\{X_{i},X_{j}\right\}}$—partial correlation between *X_i_* and *X_j_* given all others, $p_{ij}$ are the elements of the precision matrix defined as $\Omega =\Sigma^{-1}$(the inverse of the joint covariance matrix).
6. DTW distance $d_{dtw}$, which is suitable when comparing series with similar structures but shifted in time [52,53,54]. The DTW algorithm finds the smallest distance between two time series, allowing their time transformation. A local cost function $c\left(X_{ip},X_{jq}\right)=\left|x_{ip}-x_{jq}\right|,p,q=1,\ldots ,T$, is defined, and the optimal series matching involves finding a sequence of points (path) that minimizes the total cost *C*. A matching path *w* (warping path) is sought that runs through low-cost areas and avoids high costs [55,56]. The optimal warping path w* has minimum matching cost, and the distance between $X_{i}$ and $X_{j}$ is then given by
  $$d_{dtw}\left(X_{i},X_{j}\right)=C_{w*}\left(X_{i},X_{j}\right)=\min\left\{C_{w}\left(X_{i},X_{j}\right)|w\in W\right\},$$
  where W is the set of all possible warping paths.
7. Kullback–Leibler divergence (also named relative entropy) $d_{kl}$, which is a metric that quantifies the difference between two probability distributions [57]. For two continuous distributions *P* and *Q*,
  $$d_{kl}\left(P,Q\right)=\int_{-\infty }^{\infty }p\left(x\right)log\frac{p(x)}{q(x)}dx,$$
  where p and q denote the probability densities of P and Q. The nonparametric estimators for p and q were obtained in this work through kernel density estimation as
  $${\hat{f}}_{X_{t}}(x)=\frac{1}{n}\sum_{t=1}^{n}K\left(\frac{x-X_{t}}{h}\right),$$
  where K is a symmetric, positive kernel function that integrates to one, and h is the bandwidth. We used a common biweight (quadratic) kernel function, the bandwidth vector was computed by Scott’s rule of thumb, and the output was calculated over a grid of 400 points from $X_{min}$ to $X_{max}$.

### 4.3. Topological Characteristics of Networks

In order to study the structure of constructed networks, we use topological indexes of networks. The indexes used are as follows:

− average path length apl(G) (mean distance)—the mean of the lengths of the shortest paths $dist\left(u,v\right),u,v\in G$, between all pairs of vertices in the network (Wang et al., 2014) [29]
  $$apl\left(G\right)=\frac{\sum_{v,u}dist(v,u)}{\left|V\right|(\left|V\right|-1)/2}.$$

It measures the efficiency of information flow in the network and can identify a structure vulnerable to infection by negative events [58]. Since currency networks are weighted networks, the shortest path length is defined as the smallest sum of the inverse weights of the links throughout all possible paths from node u to node v;

− diameter—length of the longest shortest path between any pair of nodes
  $$D\left(G\right)=\underset{u,v}{\max}dist(v,u).$$

The smaller diameter is conducive to the transmission of information in the network [59].

In this work, we use centralization measures for the graph. Centralization is a method for calculating a graph-level centralization measure based on a node-level centrality measure. The general formula is

$$C\left(G\right)=\sum_{v}\left(\underset{w}{\max}c_{w}-c_{v}\right),$$

where $c_{v}$ is the centrality of vertex v. The centralization measures at the network level [30,60] use the following centrality concepts (at node level):

− degree centrality—indicator relating to the number of links of nodes (it measures the risk of nodes capturing all that flows through the network). The formula for degree centrality is
  $$c_{deg}\left(v\right)=\frac{number\;of\;edges\;incident\;on\;node\;v}{total\;number\;of\;nodes\;in\;the\;network-1}.$$
− closeness centrality—measure based on the average shortest path from that node to all other nodes in the network. It is defined as
  $$c_{close}\left(v\right)=\frac{1}{\sum_{u\neq v}d\left(v,u\right)},$$
  where $d\left(v,u\right)$ is the shortest path length between node v and u. Closeness centrality ranges between 0 and 1; the higher value indicates that the node is closer to all other nodes in the network on average.
− eigenvector centrality—measure of influence of a node in the network. The centrality score of a node is proportional to the sum of the centrality scores of its neighbors, with each neighbor’s score weighted by its own centrality. This means that many connections of a node are important, but also if these connections come from other important nodes. The formula for eigenvector centrality is
  $$x_{u}=\frac{1}{\lambda }\sum_{v=1}^{n}A_{uv}x_{v},$$
  where *λ* is the dominant eigenvalue of the adjacency matrix A, $A_{uv}$ is the element of the adjacency matrix, indicating whether there is a connection between nodes u and v, $x_{v}$ is the centrality score of a node v, and n is the number of nodes in the network.
− assortativity—the tendency of vertices to connect or ‘attach’ to vertices with similar properties in a graph [61]. This is a measure of homophily in a graph, which is based on some vertex labeling or values assigned to vertices (if the coefficient is high, then the connected vertices tend to have the same labels). The assortativity coefficient of a graph ranges between −1 and 1; values close to 1 indicate a very high likelihood of two vertices with the same property being connected; values close to −1 indicate a very low likelihood of two vertices with the same property being connected. Negative assortativity indicates the structure of the star network, positive—the connections of high degree nodes to high degree nodes. Assortativity describes how the vertices are connected to each other and informs on network resilience to the spread of crises.
− betweenness—shows how many times a node plays the role of a bridge on the shortest path between two other nodes; it points out the most critical vertices of the network.

Subsequently, based on the topology of the currency network, a hierarchical structure of currencies was built using the Girvan–Newman method. This method takes advantage of the observation that edges between groups usually have a higher betweenness than edges within groups [62].

## 5. Results of the Study

In the trees built during the empirical analysis, the vertices represent exchange rates, while the edge weights reflect the distances between the return series for exchange rates. Currencies are priced against each other, so the problem of choosing a numeraire arises. There is no independent numeraire, therefore a total of 40 exchange rates were analyzed against 41 base currencies. This means that a total of 1148 trees with 40 vertices each were constructed (1148 trees = 41 base currencies × 7 ways of calculating distances × 4 time periods). All these MSTs were constructed using the Prim’s algorithm.

The means and the coefficients of variation (CVs) for the topological characteristics of MSTs (trees for 41 base currencies) created using seven different distance measures, for the entire 2020–2022 period, are shown in Table 3.

Table 3 shows the topological measure values for the constructed MSTs using different distances between nodes. Weights are used for calculating weighted shortest paths. It should be noted that the average path length or closeness can be made arbitrarily large by changing only the weights (not the graph’s connectivity pattern), hence in this case the average values should not be directly compared with each other between columns. For the remaining network centralization measures, which are based on node-level centrality measures, the normalized version is computed (by dividing by the largest possible centralization value for a graph with the same number of vertices). Hence, they are comparable.

To avoid the problem of noncomparability of some measures, the coefficient of variation (CV) was used, which is the ratio of the standard deviation to the mean. It is desirable to use distance measures between nodes in the network that are more invariant over different numeraires. This proves the resistance of the MST’s structure to redefining its nodes using alternative base currencies.

It should be noted that the smallest coefficient of variation for the measures characterizes trees built using distance based on the partial correlation coefficient. Thus, this measure is characterized by a high degree of invariability.

**Figure 1:**
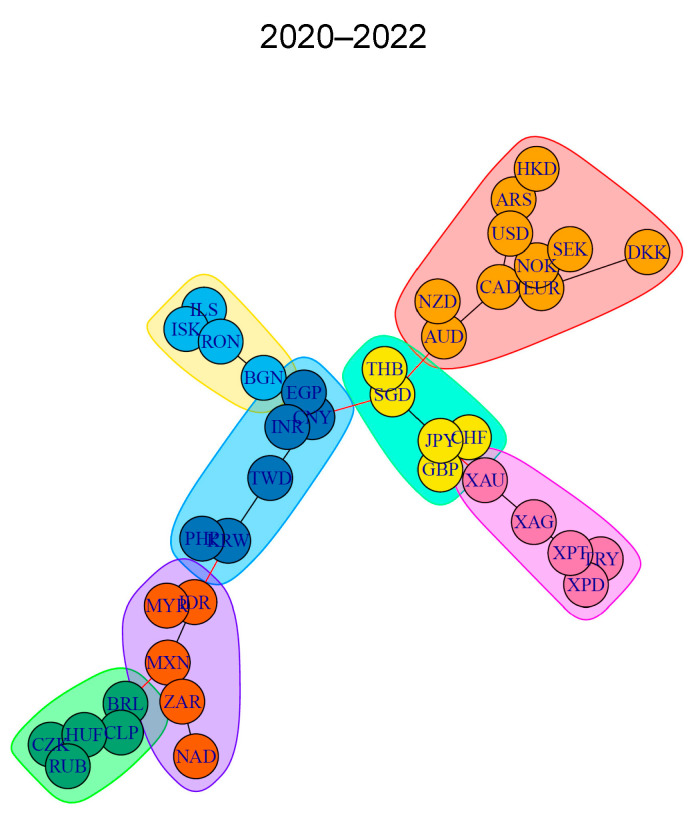
MST constructed on the basis of the partial correlation coefficient with the PLN base currency for the years 2020–2022. Source: authors’ work based on data from stooq.com (accessed on 15 January 2023).

Just for illustrative purposes, Figure 1 shows the constructed MST for the 2020–2022 period, based on the partial correlation coefficient, each time taking PLN as a base (numeraire).

Trees (MSTs) for the separate years 2020, 2021, and 2022 were analyzed in a similar way. Table 3 covers the entire analysis period, while Table 4 shows the results broken down into three separate years. The results obtained are similar. Again, MSTs built on the basis of partial correlation coefficients show the lowest variability. In all years, the use of the partial correlation coefficient resulted in the highest degree of invariability of the obtained results (see Table 4).

The results obtained confirm the thesis formulated by Basnarkov et al. (2019) [2], which states that the strength of ordinary correlations is highly dependent on the choice of base currency, while partial correlations are invariant in this aspect. Our additional calculations for the mean and CV values for the absolute values of the elements of the Pearson correlation coefficient matrices (41 matrices) and the partial correlation coefficient matrices (41) used in the construction of the MSTs again confirm this observation (see Table 5).

Thus, it can be concluded that the topological structure of currency trees with different bases for exchange rates remains similar for distances based on the partial correlation coefficient.

**Figure 2:**
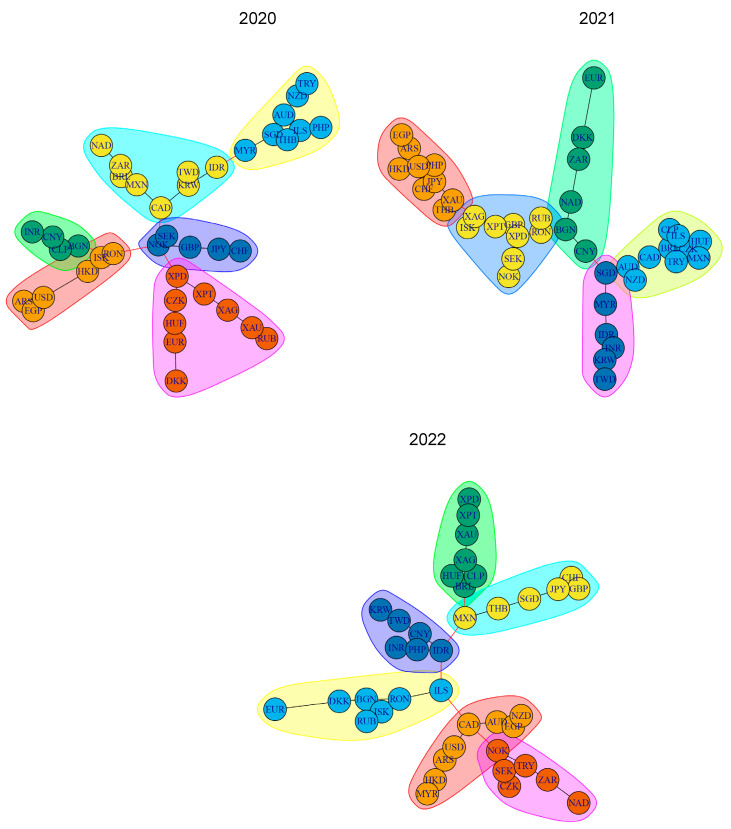
MSTs constructed on the basis of the partial correlation coefficient with the PLN base currency for the years 2020–2022, 2020, 2021, and 2022. Source: authors’ work based on data from stooq.com (accessed on 15 January 2023).

It is also worth analyzing in detail the MSTs constructed on the basis of the partial correlation coefficient, taking PLN as the base currency, for the three separate years: 2020, 2021, and 2022 (they are presented in Figure 2).

For the 2020 tree (the year of the COVID-19 pandemic outbreak), six groups of currencies were distinguished using the Girvan–Newman method. The most important of them seem to be: (1) a central cluster with NOK, SEK, GBP, JPY, and CHF, (2) a cluster with CAD, KRW, TWD, IDR, MXN, BRL, ZAR, and NAD, (3) a cluster with four metals XPD, XPT, XAG, and XAU, but also with the currencies CZK, HUF, EUR, DKK, and RUB. Noteworthy is the location of the USD, which is far from the network center. In this tree, the highest values for closeness centrality were achieved by the nodes NOK, CAD, RON, XPD, and KRW, and the highest values for betweenness were for NOK, CAD, KRW, RON, and XPD.

The MST for 2021 is characterized by five currency groups. The CNY and SGD are located close to the center, while the USD and EUR are far away. Higher closeness centrality values were recorded for ARS, AUD, BGN, BRL, and CAD, and betweenness for BGN, SGD, XPD, RON, and CNY.

For the year 2022 (the year of Russia’s invasion of Ukraine), the built MST has six currency groups. CAD, USD, AUD, and ILS are located close to the center while EUR, CHF, and GBP are located far from the center. The European cluster is worth attention, consisting of EUR, DKK, BGN, RON, ISK, and RUB (but also ILS here), and a separate Asian cluster with IDR, CNY, TWD, KRW, PHP, and INR. Higher closeness centrality was achieved for ILS, IDR, CAD, MXN, and RON, and betweenness for ILS, IDR, CAD, MXN, and BRL.

The result of comparing currency networks built for the PLN base and the USD base (using partial correlation coefficients) is also quite interesting (Table 6).

Although the obtained networks have different topological properties in subsequent years, in both crisis years—2020 (outbreak of the pandemic) and 2022 (Russian invasion of Ukraine)—the average distances between nodes decreased and graph centralization increased. Currencies became more closely related and the structure of MSTs became more correlated. This result is consistent with what was previously obtained by Miśkiewicz (2021) [34]. He showed that during crises, exchange rate correlations increase, causing more cliques and higher ranks for nodes in the network. However, our result is inconsistent with Jang et al. (2011) [25], who noted that the average correlation coefficient between currencies decreased during crises. Similar to Wang et al. (2014) [29], we found that the subsequent crisis caused by the Russian war resulted in a more central position for the USD. The war also caused the EUR to become extremely distant from the center of the network. Negative assortativity of the networks indicates the presence of stars with hubs.

## 6. Conclusions

In this study, the network topology for 37 major currencies and four metals for the year of the outbreak of the COVID-19 pandemic (2020), the time of the Russian invasion of Ukraine (2022), and the period in between (2021) are presented. Based on the distances between currency series calculated in seven different ways, minimal spanning trees (MSTs) were built for the currency network, with each currency being considered as the base currency (numeraire). The topological characteristics of the 1148 obtained trees were further analyzed.

Research question No. 1 formulated in the Introduction concerned the impact of the distance measure used on the topological structure of the currency network. Our analysis shows that different distance measures generate different trees. Despite this, the use of the partial correlation coefficient resulted in similar characteristics for trees in different databases. It can also be stated that the different distance measures used are not equally sensitive to the choice of numeraire currency, which is the answer to research question No. 2. It turned out that networks built on the basis of partial correlations provided a more adequate illustration of pairwise relationships in the foreign exchange market.

The events of 2020 and 2022 triggered changes in the topology of the currency network. As a result of the outbreak of the pandemic and Russia’s invasion of Ukraine, the average distances between tree nodes decreased and graphs centralization increased (currencies became more closely related and the MST structure was more contracted). It has been observed that the topological structure of the currency network in the periods of the pandemic crisis and the crisis caused by the Russian invasion of Ukraine differed significantly from the structure of the network in the period in between. This is the answer to research question No. 3. It should be noted that the present research, to the best of the authors’ knowledge, is the first study of the impact of the Russian war on the topological structure of the currency market network.

It is worth noticing that six out of seven dissimilarity measures employed in this paper are symmetrical with respect to the arguments. It is well-known that this property does not hold for the Kullback–Leibler relative entropy. However, it seems to be of no relevance for our investigations. We realized that although numerical values of the entropy-based dissimilarity measure for reversed arguments can be slightly different indeed, it does not change the structure of the relationships between the investigated currencies, and the constructed MSTs are practically the same.

Currencies are often pegged to other currencies due to countries’ geographic locations and economic ties, as reflected in the constructed trees. The detected structures can be useful in descriptions of the currency market, can help in constructing a stable portfolio of the foreign exchange rates, and can be a valuable tool in searching for economic factors influencing specific groups of countries.

We are fully aware that the current situation in the currency exchange markets is very volatile these days. Therefore, it would be very important to continue this kind of investigations to see if the observed nontrivial properties persist in the future, or if they are just manifestations of the specificity of the recent years. Moreover, the authors’ future research will focus on the use of network distance measures enabling the comparison of different graphs. Most of such methods described in the literature deal only with unweighted networks, while only few are able to handle weighted networks as well.

## Figures and Tables

**Table 1:** List of the currencies and metals studied.

**ARS**	Argentinian Peso	**HUF**	Hungarian Forint	**RON**	Romanian New Leo
**AUD**	Australian Dollar	**IDR**	Indonesian Rupiah	**RUB**	Russian Rubles
**BGN**	Bulgarian Lev	**ILS**	Israeli New Shekel	**SEK**	Swedish Krona
**BRL**	Brazilian Real	**INR**	Indian Rupee	**SGD**	Singaporean Dollar
**CAD**	Canadian Dollar	**ISK**	Icelandic Krona	**THB**	Thai Baht
**CHF**	Swiss Franc	**JPY**	Japanese Yen	**TRY**	Turkish Lira
**CLP**	Chilean Pesos	**KRW**	South Korean Won	**TWD**	Taiwan Dollar
**CNY**	Chinese Yuan	**MXN**	Mexican Peso	**USD**	U.S. Dollar
**CZK**	Czech Koruna	**MYR**	Malaysian Ringgit	**XAG**	Silver (ozt)
**DKK**	Danish Krone	**NAD**	Namibian Dollar	**XAU**	Gold (ozt)
**EGP**	Egyptian Pound	**NOK**	Norwegian Krone	**XPD**	Palladium (ozt)
**EUR**	Euro	**NZD**	New Zealand Dollar	**XPT**	Platinum (ozt)
**GBP**	UK Pound Sterling	**PHP**	Philippines Peso	**ZAR**	South Africa Rand
**HKD**	Hong Kong Dollar	**PLN**	Polish Zloty		

**Table 2:** Descriptive statistics for the analyzed time series measured in PLN (the whole period 1 January 2020–31 December 2022): mean, std. dev.—standard deviation, and CV—coefficient of variation. Source: authors’ work based on data from stooq.com (accessed 15 January 2023).

Currency	Mean	Std. Dev.	CV	Currency	Mean	Std. Dev.	CV	Currency	Mean	Std. Dev.	CV
**ARS**	0.044	0.010	0.230	**HUF**	0.012	0.000	0.036	**RON**	0.932	0.021	0.022
**AUD**	2.892	0.189	0.066	**IDR**	0.000	0.000	0.067	**RUB**	0.058	0.010	0.180
**BGN**	2.334	0.066	0.028	**ILS**	1.219	0.094	0.077	**SEK**	0.438	0.014	0.033
**BRL**	0.781	0.086	0.109	**INR**	0.054	0.003	0.051	**SGD**	2.977	0.209	0.070
**CAD**	3.137	0.242	0.077	**ISK**	0.031	0.002	0.065	**THB**	0.124	0.004	0.033
**CHF**	4.348	0.272	0.063	**JPY**	0.035	0.002	0.043	**TRY**	0.426	0.130	0.305
**CLP**	0.005	0.000	0.036	**KRW**	0.003	0.000	0.030	**TWD**	0.140	0.009	0.061
**CNY**	0.608	0.046	0.075	**MXN**	0.198	0.021	0.104	**USD**	4.073	0.339	0.083
**CZK**	0.179	0.010	0.055	**MYR**	0.957	0.048	0.051	**XAG**	91.001	12.552	0.138
**DKK**	0.613	0.018	0.029	**NAD**	0.257	0.018	0.071	**XAU**	7284.903	630.602	0.087
**EGP**	0.243	0.018	0.073	**NOK**	0.442	0.024	0.055	**XPD**	9057.949	1094.614	0.121
**EUR**	4.564	0.128	0.028	**NZD**	2.695	0.142	0.053	**XPT**	3976.287	462.726	0.116
**GBP**	5.268	0.235	0.045	**PHP**	0.080	0.003	0.034	**ZAR**	0.257	0.018	0.071
**HKD**	0.523	0.041	0.079								

**Table 3:** Mean and CV values for the topological characteristics of MSTs (trees for 41 base currencies) for the 2020–2022 period. In the `Characteristics’ column, all centr_* values are centralizations, i.e., the averages of the corresponding centralities over the nodes. This also applies to the following tables.

Characteristics	$d_{e}$	$d_{p}$	$d_{s}$	$d_{k}$	$d_{pcorr}$	$d_{DTW}$	$d_{kl}$
	*2020–2022*						
	Mean Values						
apl	162.277	5.833	6.385	8.436	14.575	2.292	1.443
diameter	439.147	15.785	17.500	21.688	34.392	5.767	12.253
centr_degree	0.098	0.098	0.116	0.125	0.075	0.233	0.047
centr_clo	0.241	0.241	0.233	0.242	0.181	0.284	0.098
centr_eigen	0.814	0.814	0.832	0.839	0.810	0.871	0.812
assortativity	−0.195	−0.195	−0.250	−0.236	−0.282	−0.321	−0.293
	Coefficient of Variation (CV) Values						
apl	0.378	0.378	0.352	0.269	**0.058**	0.305	0.549
diameter	0.319	0.319	0.306	0.258	**0.086**	0.297	0.410
centr_degree	0.137	0.137	0.258	0.242	**0.113**	0.431	0.287
centr_clo	0.172	0.172	0.238	0.258	**0.124**	0.320	0.229
centr_eigen	0.031	0.031	0.037	0.038	**0.019**	0.033	0.075
assortativity	−0.445	−0.445	−0.304	−0.369	**−0.172**	−0.278	−0.409

Source: authors’ work based on data from stooq.com (accessed on 15 January 2023); lowest values for CV in bold.

**Table 4:** Mean and CV values for the topological characteristics of MSTs (trees for 41 base currencies) for the years 2020, 2021, and 2022.

Characteristics	$d_{e}$	$d_{p}$	$d_{s}$	$d_{k}$	$d_{pcorr}$	$d_{DTW}$	$d_{kl}$
	*2020*						
	Mean Values						
apl	102.936	6.421	6.638	8.715	13.499	2.673	0.800
diameter	276.695	17.260	18.157	22.358	30.765	6.527	3.410
centr_degree	0.080	0.080	0.110	0.110	0.075	0.130	0.050
centr_clo	0.213	0.213	0.220	0.225	0.222	0.239	0.110
centr_eigen	0.815	0.815	0.831	0.830	0.840	0.839	0.790
assortativity	−0.145	−0.145	−0.256	−0.262	−0.102	−0.295	−0.290
	Coefficient of Variation (CV) Values						
apl	0.403	0.403	0.366	0.274	**0.079**	0.305	0.340
diameter	0.319	0.319	0.310	0.244	**0.104**	0.266	0.270
centr_degree	0.299	0.299	0.294	0.298	**0.113**	0.263	0.320
centr_clo	0.211	0.211	0.258	0.235	**0.121**	0.240	0.220
centr_eigen	0.041	0.041	0.046	0.043	**0.019**	0.050	0.080
assortativity	−0.686	−0.686	−0.392	−0.322	−0.769	**−0.265**	−0.370
	*2021*						
	Mean Values						
apl	97.806	6.089	6.490	8.632	15.788	3.125	0.580
diameter	271.857	16.925	18.024	22.531	38.599	8.000	4.520
centr_degree	0.131	0.131	0.122	0.129	0.075	0.112	0.060
centr_clo	0.239	0.239	0.230	0.232	0.142	0.215	0.120
centr_eigen	0.841	0.841	0.836	0.843	0.801	0.835	0.810
assortativity	−0.312	−0.312	−0.302	−0.284	−0.366	−0.299	−0.300
	Coefficient of Variation (CV) Values						
apl	0.395	0.395	0.376	0.285	**0.063**	0.358	0.250
diameter	0.311	0.311	0.323	0.275	**0.101**	0.336	0.270
centr_degree	0.241	0.241	0.279	0.254	**0.113**	0.248	0.300
centr_clo	0.241	0.241	0.236	0.259	**0.170**	0.291	0.220
centr_eigen	**0.040**	**0.040**	0.043	0.044	0.056	0.052	0.060
assortativity	−0.233	−0.233	−0.286	−0.274	**−0.160**	−0.351	−0.430
	*2022*						
	Mean Values						
apl	92.492	5.769	6.042	8.088	13.122	2.743	0.920
diameter	254.037	15.846	16.317	20.270	27.875	6.442	8.120
centr_degree	0.119	0.119	0.119	0.123	0.057	0.124	0.060
centr_clo	0.250	0.250	0.255	0.264	0.211	0.242	0.110
centr_eigen	0.838	0.838	0.840	0.842	0.764	0.835	0.810
assortativity	−0.299	−0.299	−0.214	−0.214	0.008	−0.327	−0.330
	Coefficient of Variation (CV) Values						
apl	0.387	0.387	0.339	0.245	**0.042**	0.335	0.490
diameter	0.367	0.367	0.300	0.237	**0.101**	0.329	0.440
centr_degree	0.239	0.239	0.264	0.248	**0.172**	0.257	0.240
centr_clo	0.242	0.242	0.211	0.176	**0.058**	0.240	0.220
centr_eigen	0.044	0.044	0.036	**0.027**	0.035	0.053	0.070
assortativity	**−0.224**	**−0.224**	−0.465	−0.391	9.087	−0.266	−0.320

Source: authors’ work based on data from stooq.com (accessed on 15 January 2023); lowest values for CV in bold.

**Table 5:** Mean and CV values for the averaged absolute values of the elements of the Pearson correlation coefficient matrices (41 matrices) and the partial correlation coefficient matrices (41) used in the construction of the MSTs.

Time Period	$\left|\rho_{p}\right|$		$\left|\rho_{pcorr}\right|$	
	Mean	CV	Mean	CV
2020–2022	0.481	0.397	0.077	0.007
2021	0.484	0.369	0.101	0.006
2022	0.480	0.392	0.093	0.007
2023	0.487	0.374	0.095	0.006

Source: authors’ work based on data from stooq.com (accessed on 15 January 2023).

**Table 6:** Selected topological characteristics for MSTs based on PLN and USD.

Base PLN	*2020–2022*	*2020*	*2021*	*2022*
apl	14.183	13.405	16.077	12.934
diameter	32.420	30.344	38.505	27.305
centr_degree	0.078	0.078	0.078	0.053
centr_clo	0.186	0.221	0.135	0.217
centr_eigen	0.804	0.846	0.782	0.756
assortativity	−0.311	−0.072	−0.419	0.006
**Base USD**	***2020–2022***	***2020***	***2021***	***2022***
apl	14.432	13.649	15.912	13.184
diameter	34.519	30.344	40.760	27.305
centr_degree	0.078	0.078	0.078	0.078
centr_clo	0.186	0.221	0.141	0.220
centr_eigen	0.827	0.845	0.787	0.824
assortativity	−0.202	−0.109	−0.357	−0.082

Source: authors’ work based on data from stooq.com (accessed on 15 January 2023).

## Data Availability

The data presented in this study are available on request from the corresponding author.
